# A Case of Hyperpyrexia Following Measles, Treated Successfully by the Wet-Pack and Cold Douche

**Published:** 1885-06

**Authors:** Christopher Elliott

**Affiliations:** Physician to the Bristol Hospital for Sick Children


### Clinical Records.

### A CASE OF HYPERPYREXIA FOLLOWING MEASLES, TREATED SUCCESSFULLY BY THE WET-PACK AND COLD DOUCHE. By CHRISTOPHER ELLIOTT, B.A., M.D., Physician to the Bristol Hospital for Sick Children.

THE treatment of Hyperpyrexia by the cold bath has had many able advocates from time to time, more especially in connection with enteric fever; but beyond the record of a few isolated cases in the medical journals (so far as I can judge from the literature on the subject), not much attention has been paid to its treatment by the wet-pack. Apart from the fact that those who have had most experience with the cold bath are not agreed amongst themselves as to its safety or value, there is one drawback to its general adoption in private practice; viz., that a sufficiently large bath is not always to be obtained in the houses of our patients. Materials for a wet-pack, however, can be usually found; for all we require is a macintosh or a large piece of indiarubber sheeting, one or two blankets, a sheet, and a can of cold water. When the bed is a wide one, these may be placed in it by the side of the patient, and, if proper care be taken, he can be easily transferred to and from the pack without wetting the bed. Besides having a diaphoretic action on the skin, the pack calms the nervous centres, subduing restlessness, and causing sleep; but, notwithstanding that it acts thus, I did not find that it could be relied upon by itself to cause a rapid and decided reduction of temperature, and for this reason I am in favour of following up its application with the cold douche. The nervous shock, which Dr. Cayley considers to be an important element in the cold bath treatment, can be communicated as efficiently in this way, and I believe with greater safety to the patient, as well as with far less trouble to the physician.

Another advantage of this combined method is, that we can dispense with the administration of medicines, which is no slight point gained, when the stomach is intolerant of almost everything that is put into it. The case I am about to bring forward would be more correctly described as one of measles complicated with pneumonia, internal otitis, meningitis, and hyperpyrexia. The pyrexia lasted over six weeks, during which time the temperature was twice above 107°, twice 106°, fifteen times between 106° and 105°, and altogether fifty-eight times above 103°. Without going into all the details, the case briefly was as follows :

M. E., æt. 5 years, was taken ill on March 12th, 1884, being one of five in a household attacked with measles. The disease appeared to be taking a mild course, as on the 17th (the fifth day of illness) the temperature was subnormal; but next day my patient complained of pain in the right ear, and on the following day I detected slight dulness, with fine crepitus, at the base of the right lung. The mischief in the lung steadily increased, until quite two-thirds of it was involved, and on March 21st the evening temperature was 105·2°. On the 22nd she vomited several times in the course of the day, and in the evening complained of stiffness in the neck. T. 105·6°,

R. 52. Next evening the temperature was again 105·6°, and there were rigors, and at the same time the child called out that she saw "two mothers and two nurses!" The double vision did not, however, last long, as it had quite passed away when I arrived shortly after, and I could not then detect any squint to account for it. On the following day there was a slight discharge of pus and blood from the right ear, but it ceased the next day. On March 26th the patient complained of nausea and chilliness, and again on the 29th.

On March 31st, as the temperature had been rising above 105° for the past few days, and having failed to reduce it by the administration of quinine, either by the mouth or in enema, as suggested to me by Dr. E. L. Fox, I determined to try if the wet-pack would assist me. The condition of the lung was all this time unchanged, and there were present also symptoms pointing to cerebral irritation, such as great fractiousness, sleeplessness, deafness, and frequent vomiting, followed later on by a well-marked internal strabismus of the right eye. I confess I felt somewhat timid at the first trial of the pack, and kept it on for only forty-five minutes; but although the period was too short, and there was no immediate reduction of the temperature, yet, five hours after, it had fallen from 105·2° to 103·2°, and there was less restlessness.

Next day, April 1st, the temperature being again 105·2°, I put my patient into the pack, and, without removing her, I ascertained at the end of an hour that the temperature was ·2° higher. I now administered four grains of quinine, and kept her in the pack for another hour. At the end of that time she vomited, but the temperature had fallen to 104·2° (=1·2°). Eight hours after it was 103·2°, and all next day did not rise above 103·4°.

On April 3rd, at 4.45 a.m., the patient had a severe rigor, and vomited. T. 107·2°. Half an hour after, it had fallen to 105·2°; but nevertheless I employed the pack for an hour, and two hours after the temperature had fallen to 100·8°. In the evening, at nine p.m., the temperature had again risen to 105·4°, and I re-applied the pack for forty-five minutes. Three-quarters of an hour after the temperature had dropped 1·5°, and next morning was only 100·4°.

On April 5th, at midnight, the temperature was 106°, and, having been summoned, I put my patient immediately into the pack, and administered three grains of quinine. At the end of an hour she was taken out, having slept nearly all the time, and was then given some liquid nourishment, but immediately vomited it. However, the temperature had fallen over 2° (103·8°), and, notwithstanding that there was troublesome sickness for the next few hours, it continued to fall, and was 100·4° next morning at 10.0 a.m., nine hours after the pack.

In the evening of April 6th, however, it was again 106° at 10 p.m., and without delay I applied the pack. At the end of an hour I took the temperature and found it to be 107·2°; and as the quinine had caused such intense sickness the last time it was given, I now decided to keep my patient in the pack for two hours longer, and at the end of that time to apply cold affusion. This I did with a large sponge, douching the body from head to foot with cold water. I next placed her in warm blankets, and, taking the temperature, I ascertained that it had fallen nearly five degrees, being now 102·4°. This was followed by a sound sleep of seven hours duration, and next day my patient's condition appeared changed for the better. On examining the chest, I found that the lung had almost entirely cleared up; but the squint was still very decided. On the following day a little pus began to flow from the right ear; but, although it continued for some time, it was never much in quantity. For over a fortnight longer the temperature fluctuated a good deal, and on two occasions gave me some anxiety, as it rose above 105°; but I did not find it necessary to employ the pack or douche again. Convalescence was also further delayed by the left foot and ankle-joint becoming painful and swollen; but, keeping the limb at rest on a splint with a foot-piece, this was got under, and ultimately my patient made an excellent recovery, losing all trace of the squint, and without any deafness or lameness.

